# Association of multilocus sequencing types and antimicrobial resistance profiles of methicillin-resistant *Mammaliicoccus sciuri* in animals in Southern Thailand

**DOI:** 10.14202/vetworld.2023.291-295

**Published:** 2023-02-15

**Authors:** Kanpapat Boonchuay, Narin Sontigun, Tuempong Wongtawan, Punpichaya Fungwithaya

**Affiliations:** 1 Akkhraratchakumari Veterinary College, Walailak University, Nakhon Si Thammarat 80160, Thailand; 2 One Health Research Center, Walailak University, Nakhon Si Thammarat 80160, Thailand; 3 Centre of Excellence Research for Melioidosis and Other Microorganisms, Walailak University, Nakhon Si Thammarat 80160, Thailand

**Keywords:** antibiogram, *Mammaliicoccus sciuri*, methicillin-resistant bacteria, multilocus sequencing types, resistant gene

## Abstract

**Background and Aim:**

*Mammaliicoccus sciuri*, formerly known as *Staphylococcus sciuri*, is an opportunistic pathogen in the environment, human and animal mucosa, and skin. Although this pathogen is becoming more resistant to drugs and harmful to animals and humans, basic knowledge of this pathogen remains limited. This study aimed to investigate a new multilocus sequencing type (MLST) related to the antibiotic resistance pattern of *M. sciuri* from animals in southern Thailand.

**Materials and Methods:**

We used 11 methicillin-resistant *M. sciuri* (MRMS) isolates in this study which were obtained from six horses, four cows, and one chicken of the previous study. Antimicrobial resistance (AMR) was re-evaluated based on the minimum inhibitory concentration using the VITEK^®^ 2 automated system. Three AMR genes were examined, namely *mecA*, *mecC*, and *blaZ*. Staphylococcal chromosomal cassette *mec* (SCC*mec*) gene detection was performed through the multiplex polymerase chain reaction (PCR). Internal segments of the seven housekeeping genes, *ack*, *aroE*, *ftsZ*, *glpK*, *gmk*, *pta1*, and *tpiA*, were used for multilocus sequence typing. The population of resistant bacteria and the types of multidrug-resistant, extensively drug-resistant, and pandemic drug-resistant bacteria were classified through descriptive analysis.

**Results:**

*mecA* and *blaZ* genes were detected in all isolates; however, the *mecC* gene was not observed in any isolate based on the PCR results. All MRMS isolates revealed a non-typable SCC*mec*. Seven MLSTs (71, 81, 120, 121, 122, 199, and 200) were identified in this study.

**Conclusion:**

The characteristics of MRMS in Southern Thailand were variable, particularly in cattle and horses. The antibiogram and SCC*mec* types of this pathogen remain concerns with regard to antibiotic-resistant gene transmission among *Staphylococcus* and *Mammaliicoccus* species. All MLSTs in Thailand revealed the distribution among clones in Asia, including the virulence of a zoonotic clone in Southern Thailand.

## Introduction

*Mammaliicoccus* spp. has been reassigned from *Staphylococcus* spp. in 2020 [1]. This genus is comprises of *Staphylococcus sciuri*, *Staphylococcus fleurettii*, *Staphylococcus lentus*, *Staphylococcus stepanovicii*, and *Staphylococcus vitulinus* [1]. *Mammaliicoccus sciuri* (or *S. sciuri*) is a gram-positive and coagulase-negative cocci commonly found in the environment, humans, and animals [2–4]. Furthermore, it is an opportunistic bacterium capable of causing severe human infections [5, 6]. *Mammaliicoccus sciuri* has been discovered in various healthy and unhealthy animals, particularly ruminants [3–5, 7, 8]. However, the transmission of *M. sciuri* between humans, animals, and the environment has not been reported. *Mammaliicoccus sciuri* exists in the environment, especially bedding in dairy farms [2].

Multidrug-resistant (MDR) bacteria are an important problem in hospitals because they resist several antibiotic drugs [5]. Methicillin-resistant staphylococci (MRS) are harmful bacteria that cause nosocomial infections in humans and animals [3, 5]. They can carry MDR genes on the large mobile genetic element, staphylococcal chromosomal cassette *mec* (SCC*mec*), which exhibits intra-and inter-species transfer between staphylococci and mammaliicocci [9, 10]. In this element, the resistance genes can be transferred from one bacterium to others, which leads to problems with antibiotic drugs in both human and veterinary hospitals [11]. The *bla*Z gene encodes a penicillinase enzyme that resists beta-lactam antibiotics [12]. In Thailand, most staphylococci carry the *bla*Z gene, which makes penicillin ineffective for staphylococci elimination [7, 13]. Methicillin-resistant *M. sciuri* (MRMS) or methicillin-resistant *S. sciuri* has been reported in humans, animals, and the environment [2, 4, 7, 14–16], of which healthy animals act as carriers of this pathogen [7]. Methicillin-resistant *M. sciuri* has been reported in healthy animals in Thailand since 2022 [7].

Multilocus sequencing type (MLST) is developed from seven housekeeping genes specific to each bacterial species [17]. At present, 342 MLST schemes of *M. sciuri* have been discovered in several countries, including China, Austria, and Canada [18, 19]. The sequence type (ST) of *M. sciuri* is classified into seven housekeeping genes: *ack*, *aroE*, *ftsZ*, *glpK*, *gmk*, *pta1*, and *tpiA* [4]. Most MLSTs of *M. sciuri* have been reported in Europe (183 isolates), Asia (97 isolates), and North America (36 isolates) [19] and were retrieved from unknown diseases, carriers, and mastitis [19]. In Thailand, only one MLST of *M. sciuri* has been reported and found in food, with no reports from animals or humans [19]. Therefore, true sources of *M. sciuri* have not been described in animals in Southern Thailand.

This study aimed to investigate a new MLST related to the antibiotic resistance pattern of *M. sciuri* in animals in Southern Thailand.

## Materials and Methods

### Ethical approval

This study used samples from a previous study approved by the Institutional Animal Care and Use Committee of Walailak University (Project number WU-AICUC-63-023).

### Study period and location

The study was conducted from January to May 2021. Samples were collected from animals that lived in nearby areas of Walailak University (within 1 km). The samples were processed at Research Institute for Health Sciences, Walailak University.

### Stock isolation

From the 22 MRMS isolates in a previous study, 11 MRMS isolates were selected and retrieved from frozen (−80°C) stock [7]. The selection criteria included isolates from animals that lived in nearby areas of Walailak University (within 1 km) and had been defined as MRMS in a previous study. These isolates were obtained from six horses, four cows, and one chicken [7]. All isolates were reidentified using the VITEK^®^ 2 card for Gram-positive Organisms in conjunction with the VITEK^®^ 2 COMPACT machine (bioMérieux, Marcy l’Etoile, France) before use.

### Antimicrobial resistance (AMR) profile, AMR gene detection, and *SCCmec* typing

All samples were examined for AMR patterns through a minimum inhibitory concentration (MIC) - based automated system, the VITEK^®^ 2 AST-GN80 test kit cards, and the VITEK^®^ 2 COMPACT machine (bioMérieux) [20, 21]. The MIC results were interpreted using an advanced Expert System™ based on the global Clinical and Laboratory Standards Institute and natural resistance guidelines, as in a previous study [7]. The following antimicrobial drugs were included in the VITEK^®^ 2 AST-GN80 test: Benzylpenicillin, oxacillin, cefalotin (CEF), cefovecin (CEC), ceftiofur (CET), kanamycin (KAN), enrofloxacin (ENR), marbofloxacin (MAR), pradofloxacin, erythromycin, clindamycin (CLI), doxycycline, tetracycline (TCN), nitrofurantoin (NFT), chloramphenicol, and sulfamethoxazole/trimethoprim (SXT). Three AMR genes were re-examined, namely *mecA*, *mecC*, and *blaZ* [21–23]. Polymerase chain reaction (PCR) was performed with the primers used in a previous study [7]. Staphylococcal chromosomal cassette *mec* gene detection was performed through multiplex PCR [24].

### Multilocus sequencing type

Primers for several potential housekeeping gene fragments (*ack*, *aroE*, *ftsZ*, *glpK*, *gmk*, *pta1*, and *tpiA*) were obtained from Schauer *et al*. [4], and gene detection was performed in all samples through single PCR. The PCR profile was as follows: 94°C for 300 s, followed by 30 cycles each of denaturation at 94°C for 30 s, annealing at 55°C for 30 s (except for *ftsZ* [53°C]), and extension at 72°C for 1 min, and a final extension at 72°C for 5 min. The PCR products were purified by Tiangen Biotech (Beijing) Co., Ltd., (China) and sent for sequencing (Ward Medic Ltd., Part, Bangkok, Thailand). The sequencing results were analyzed using PubMLST [25]. A new ST is required to validate sequencing data before it can be published in PubMLST [25].

### Statistical analysis

The population of MDR bacteria was expressed as a proportion. The types of MDR, extensively drug-resistant (XDR), and pandemic drug-resistant bacteria were classified according to a study by Magiorakos *et al*. [26]. Staphylococcal chromosomal cassette *mec* typing was performed as described by Kondo *et al*. [24].

## Results

### AMR profile and genes

All bacterial profiles are shown in Table-1, in which the samples were resistant to 11 drugs. Highly resistant drugs (>50% resistance rate) were CEC (72.7%), CET (54.5%), CLI (72.7%), and TCN (54.5%). The chicken sample was resistant to KAN and SXT; nine were MDR or XDR bacteria (9/11, 81.81%). Only one MRMS isolate from chicken resisted 10 antibiotic drugs, whereas the other isolates from horses and cows resisted at least two and four antibiotic drugs, respectively. All samples were sensitive to CEF, gentamicin, neomycin, ENR, MAR, and NFT. Clindamycin resistance was not observed in this study.

**Table-1:** Results of 11 MRMS with 11 common veterinary drugs used in Thailand.

No.	Host	Resistant antibiotics	SCC*mec* typing			ST

			*ccr* type	*mec* type	SCC*mec*	
1	Chicken	BPC/CET/CEC/KAN/PRA/ ERY/CLI/DOX/TCN/SXT	*ccr*C	NT	V	71
2	Horse	BPC/CET/CLI	NT	classB	NT	120
3	Cow	BPC/CET/PRA/CLI	*ccr*C	classB	NT	81
4	Cow	BPC/CLI	NT	classB	NT	200
5	Cow	BPC/CLI	NT	classB	NT	120
6	Cow	BPC/CLI	*ccr*C	classB	NT	121
7	Horse	BPC/CET/CEC/PRA/DOX/TCN/CAP	NT	NT	NT	71
8	Horse	BPC/CET/CEC/PRA/DOX/TCN/CAP	*ccr*C	NT	V	71
9	Horse	BPC/CET/CEC/PRA/TCN	*ccr*C	NT	V	199
10	Horse	BPC/CET/CEC/CLI/TCN	NT	NT	NT	122
11	Horse	BPC/CET/CEC/CLI/TCN	NT	classA	NT	122

BPC=Benzylpenicillin, OXA=Oxacillin, CET=Cefalotin, CEC=Cefovecin, CET=Ceftiofur, KAN=Kanamycin, ENR=Enrofloxacin, MAR=Marbofloxacin, PRA=Pradofloxacin, ERY=Erythromycin, CLI=Clindamycin, DOX=Doxycycline, TCN=Tetracycline, NFT=Nitrofurantoin, CAP=Chloramphenicol, SXT=Sulfamethoxazole/Trimethoprim, NT=Non typeable, MRMS=Methicillin-resistant *Mammaliicoccus sciuri*

All samples harbored AMR genes, including *mecA* and *blaZ* genes but not the *mecC* gene. The *ccr*, *mec*, and SCC*mec* types are shown in Table-1. All MRMS isolates showed a non-typable (NT) SCC*mec*. Five harbored *ccrC* on their large mobile genetic element (5/11, 45.45%), whereas the other isolates did not harbor *ccr* types (6/11, 54.54%). Five NT, one class A, and five class B *mec* types were observed. Only two samples showed both NT *ccr* and *mec* types; three were interpreted as SCC*mec* type V following the primer publication.

### Multilocus sequencing type

Seven MLSTs were found in this study (Table-1): 71, 81, 120, 121, 122, 199, and 200. Five were discovered as new STs (ST 120, 121, 122, 199, and 200). The clones from cattle were more varied in STs than those from horses. The most predominant clone was ST71 (3/11, 27.27%). This clone resisted at least seven antibiotic drugs and acted as MDR and XDR strains. Five antibiotic-resistant clones presented ST122 and ST199. ST81 was resistant to four antibiotic drugs, whereas ST120, ST121, and ST200 were sensitive to most antibiotic drugs.

The variables of the seven housekeeping gene fragments (*ack*, *aroE*, *ftsZ*, *glpK*, *gmk*, *pta1*, and *tpiA*) in this study are shown in Table-1. Four housekeeping gene fragments (*ack, glpK*, *gmk*, and *tpiA*) demonstrated seven different allele types. The *pta1* and *ftsZ* genes showed the highest and lowest number of different allele types, respectively.

## Discussion

Methicillin-resistant *M. sciuri* is a pathogen s found in humans, animals, and the environment. This study revealed five new STs in the world from MRMS isolates from animals in Southern Thailand. Two STs of MRMS observed in the study are related to the clone in Asia, specifically China and Bangladesh [19]. The AMR profiles of MRMS in this study varied with resistance to at least two categories of antibiotic drugs. Several isolates were either MDR or XDR, which can threaten animal and human health. All isolates harbored the *blaZ* gene with NT SCC*mec*. The ST related to the Chinese clone showed high virulence, and each isolate resisted more than six antibiotics [4, 19].

MRMS has been reported in several animal species in various countries [2, 3, 7]. Beta-lactam antibiotics are commonly used in veterinary medicine in Thailand [27]. This study revealed that all MRMS isolates in Thailand harbored *blaZ* genes with resistance to at least two antibiotic drugs, which is in accordance with a previous study in Thailand [7]. In general, *blaZ* genes are found on the chromosome or plasmids of staphylococci, which are transferred among staphylococci through phage transduction [11, 12]. Phages that carry *blaZ* genes may be widely distributed in the environment [28]. Based on the results, the first-line antibiotic drugs for animal treatment should be beta-lactam antibiotics with a penicillinase inhibitor.

Various SCC*mec* types have been identified in MRS and MRM [4, 7, 29]. The SCC*mec* variable type might depend on the study area and phage type in the area [4, 28, 29]. Because of limitations in primer interpretation, the SCC*mec* type V found in this study could be V, VII, or XII [29, 30]. The accurate SCC*mec* type may be determined in future whole-genome sequencing studies.

Since 2020, MLSTs of MRMS have been reported in several countries, including Austria, China, and Canada [19]. Based on our results, MRMS ST71 was one of the most popular clones in Thailand. This clone was discovered for the first time in China in an unreported [19]. In Thailand, this pathogen clone has been shown in horses and chickens to resist more than seven antibiotic drugs. Therefore, we suggest that this clone is of concern in Thailand. Another clone of concern is ST81, which has been reported in a neonatal infection in Bangladesh [6]. In this case, zoonotic infections must be classified and monitored to protect humans in Thailand. At present, five new STs have been reported exclusively in Thailand. The virulence and zoonotic abilities of these new STs remain unknown. The resistance of the five new clones was lower than those of ST71 and 81. However, MRMS clones in Thailand must be monitored to tailor antibiotic use in Thailand.

## Conclusion

The characteristics of MRMS in Southern Thailand are variable, particularly for cattle and horses. The antibiogram and SCC*mec* types of this pathogen remain a concern with regard to antibiotic-resistance gene transmission among *Staphylococcus* and *Mammaliicoccus* species. All MLSTs in Thailand revealed the distribution among clones in Asia, including the virulence of zoonotic clones in Thailand.

## Authors’ Contributions

KB: Laboratory works, data analysis, methodology, validation, formal analysis, investigation, and resources. NS: Sample collection and writing−review and editing. TW: Writing−original draft preparation and writing−review and editing. PF: Conceptualization, funding acquisition, methodology, formal analysis, investigation, resources, data curation, writing−original draft preparation, and writing−review and editing. All authors have read and approved the final manuscript.
